# Optimized gradient of lyophilized platelet-rich plasma in biomimetic 3D-printed triphasic scaffold based on alginate and gelatin for osteochondral tissue engineering

**DOI:** 10.1038/s41598-026-37615-7

**Published:** 2026-01-27

**Authors:** Faezeh Ghobadi, Maryam Mohammadi, Rooja Kalantarzadeh, Arezoo Ashrafnia Menarbazari, Jila Majidi, Ehsan Lotfi, Shokoufeh Borhan, Yousef Fatahi, Narendra Pal Singh Chauhan, Ghazaleh Salehi, Sara Simorgh

**Affiliations:** 1 Cellular and Molecular Research Center, Iran University of Medical Sciences, Tehran, Iran; 2 Department of Tissue Engineering and Regenerative Medicine, Faculty of Advanced Technologies in Medicine, Iran University of Medical Sciences, Tehran, Iran; 3 Department of Medical Biotechnology, Faculty of Allied Medical Sciences, Iran University of Medical Sciences, Tehran, Iran; 4 Department of Materials, Chemical and Polymer Engineering, Buein Zahra Technical University, Buein Zahra, Qazvin Iran; 5 Nanotechnology Research Centre, Faculty of Pharmacy, Tehran University of Medical Sciences, Tehran, Iran; 6 Department of Pharmaceutical Nanotechnology, Faculty of Pharmacy, Tehran University of Medical Sciences, Tehran, Iran; 7 Department of Chemistry, Faculty of Science, Bhupal Nobles’ University, Udaipur, 313001 Rajasthan India

**Keywords:** Osteochondral tissue engineering, Biomimetic 3D-printed scaffold, Lyophilized platelet-rich plasma, Biotechnology, Materials science, Medical research, Stem cells

## Abstract

This study developed a 3D-printed, triphasic (subchondral bone, calcified, and articular cartilage) scaffold using biological macromolecule-based bioinks to support the chondrogenic differentiation of bone marrow-derived mesenchymal stem cells (BM-MSCs). A subchondral bone layer was formed by blending various concentrations of graphene oxide (GO) (1% and 2% w/w) into an alginate (Alg) and gelatin (Gel) bioink, two natural biopolymers known for their biocompatibility and biodegradability. Following mechanical and biocompatibility assessments, the 1% GO concentration was selected and applied consistently through the subchondral and calcified cartilage layers. In contrast, the gradient of lyophilized platelet-rich plasma (PRP) powder was adjusted to 1%, 2%, and 3% (w/v) to more accurately replicate the characteristics of calcified and articular cartilage. Triphasic scaffolds with different PRP gradients were evaluated for water absorption, biodegradability, rheological behavior, stem cell viability, and chondroinductive activity. The results indicated that 3D-printed triphasic scaffolds containing 1% or 2% PRP exhibited favorable biomechanical properties, with no significant differences between the two concentrations. However, scaffolds with 2% PRP facilitated the attachment, proliferation, and survival of BM-MSCs, as indicated by an increase in the expression of cartilage-related genes and enhanced production of glycosaminoglycan (GAG), as confirmed through real-time PCR and Alcian Blue staining, respectively.

## Introduction

 Osteochondral (OC) tissue includes three key components: articular cartilage, calcified layer, and subchondral bone. Each area has a specific function for managing the mechanical forces during joint activities^1^. Repairing OC defects requires simultaneous subchondral bone repair and cartilage regeneration^2^. Strategies for tissue engineering (TE) of cartilage and bone often involve encapsulating cells or growth factors (GFs) within various types of scaffolds, such as hydrogels^3^. Recent advances in biological repair methods have resulted in the development of scaffolds with complex structures and distinct biomechanical properties. These advances provide essential mechanical support for cellular activities and enhance osteochondral tissue engineering (OCT)^4^. Additive manufacturing, particularly 3D printing, has become increasingly popular for producing customized OC scaffolds. The application of multilayer 3D printing, which integrates bioactive agents with different gradients, has made it possible to create multiphasic structures with accurate tissue modeling, facilitating the development of bioinspired scaffolds^5^. This technology lets the construction of scaffolds with tailored sizes, pore structures, and mechanical characteristics using synthetic and natural biomaterials as bioinks^6^.

Alginate (Alg) is a biocompatible natural polymer frequently utilized for creating scaffolds due to its bioactivity and ease of modification through cross-linking. Alg’s ability to change from a liquid to a rigid hydrogel makes it particularly useful for 3D printing^7^. However, its limited biocompatibility and bioactivity have led to the incorporation of other bioactive polymers, such as gelatin (Gel), to mimic extracellular matrix (ECM) properties^8^. Alg-Gel blends promote enhanced cell proliferation and outperform pure Alg scaffolds, making them superior choices for biomedical applications^9^. Although hydrogels offer several benefits, their limited mechanical strength and poor processability pose significant challenges in 3D printing applications. To address these limitations, researchers have created reinforced hydrogels incorporating nanomaterials such as hydroxyapatite, clay, and graphene oxide (GO)^10^. Among these nanomaterials, GO is particularly notable for its bioactivity and has demonstrated significant promise in bone regeneration, especially when used in conjunction with the Alg-Gel hydrogel. Incorporating GO into Alg-Gel scaffolds enhances their mechanical stability and biological efficacy, leading to increased bone mineralization^11^. Conversely, the application of GFs, including those present in platelet-rich plasma (PRP), can enhance chondrogenesis and promote cartilage ECM synthesis. PRP is particularly rich in various GFs, such as vascular endothelial growth factor (VEGF), platelet-derived growth factor (PDGF), transforming growth factor beta (TGF-β), basic fibroblast growth factor (BFGF), and epidermal growth factor (EGF)^12^. These GFs play a pivotal role in enhancing the regenerative potential of OCTE strategies, especially by promoting the differentiation of bone marrow-derived mesenchymal stem cells (BM-MSCs) into chondroblasts and supporting cartilage ECM production^13^. Lyophilization allows medical professionals to administer an exact dosage of GFs. Research conducted on animals has demonstrated that lyophilized-PRP implants are both biodegradable and biocompatible and are effective in promoting cartilage repair^14^.

**Fig. 1:**
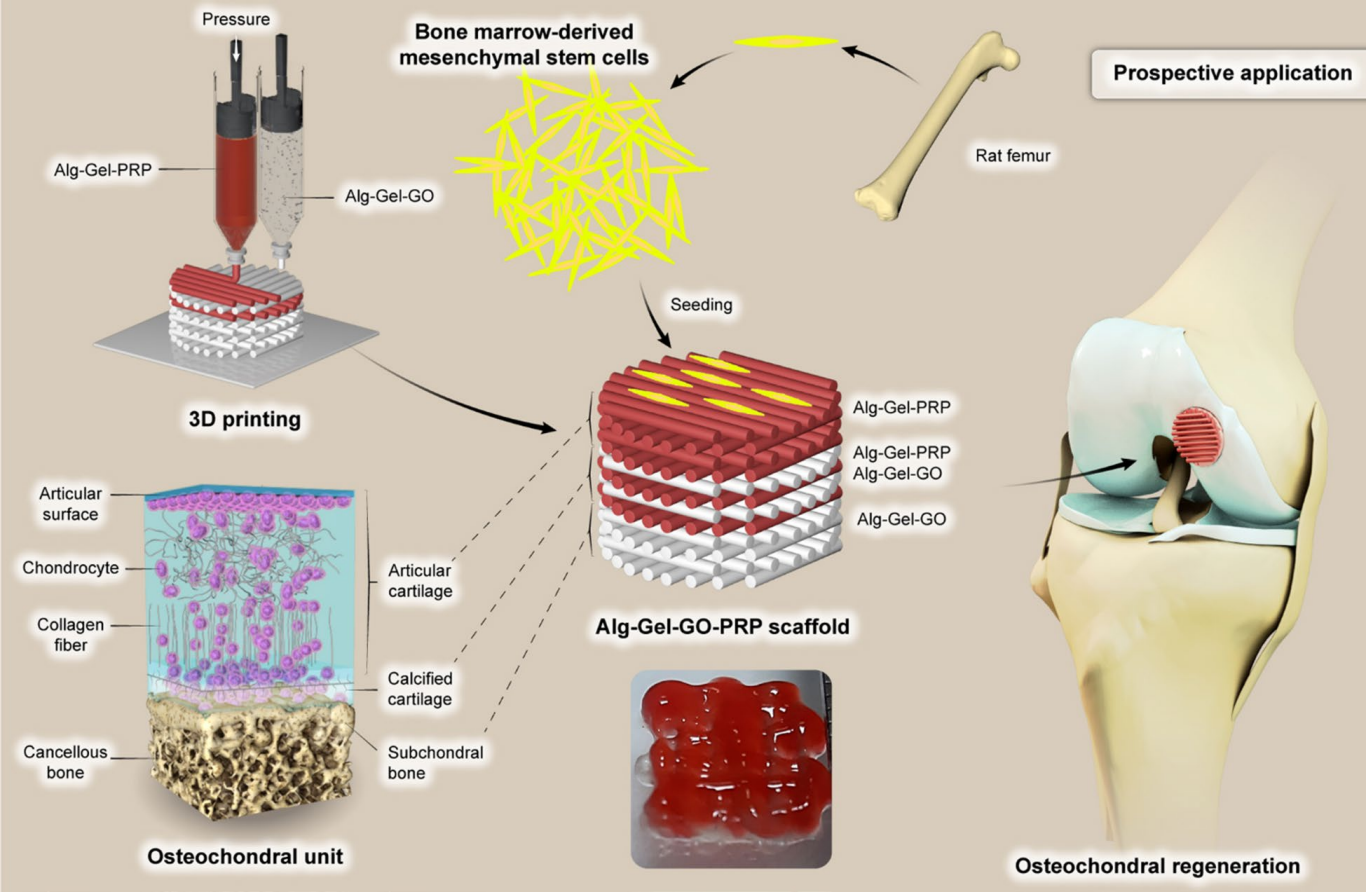
Schematic overview of the research.

The identified research gap arises from a lack of studies focused on fabricating 3D-printed, specifically triphasic, bioactive scaffolds with GO and lyophilized-PRP. Although there is existing research on 3D-printed scaffolds that use GO to enhance mechanical properties and PRP to promote cell proliferation and differentiation, the synergistic combination of these materials for biomimetic OC ECM in a single, graded triphasic scaffold has not been thoroughly explored. The objective of this project was to create gradient biocompatible triphasic multilayered scaffolds characterized by unique mechanical and biological properties. The top layers, designed to mimic articular cartilage, are composed of a blend of Alg and Gel bioink, along with an optimal concentration of lyophilized PRP powder. The middle layers consist of a layer-by-layer combination of the Alg-Gel with the optimal PRP dosage and the Alg-Gel with the optimal GO dosage to replicate the characteristics of calcified cartilage. The bottom layers, representing the subchondral bone, incorporate Alg-Gel bioink with an optimal GO dosage. To the best of our knowledge, this study is the first to develop a functionally graded OC scaffold using triphasic Alg-Gel-GO-PRP bioink via extrusion-based 3D printing. We then evaluated the mechanical and biological performance of this chondroinductive 3D-printed triphasic scaffold for in vitro chondrogenesis of BM-MSCs (Fig. 1).

## Materials and methods

### Materials

Dulbecco’s Modified Eagle’s Medium (DMEM), fetal bovine serum (FBS), penicillin-streptomycin (pen/strep), and 0.25% trypsin-EDTA were procured from Gibco (USA). The constructs were cultured in a chondrogenic medium composed of high-glucose DMEM, sodium pyruvate, L-proline, L-ascorbic acid-2-phosphate, insulin transferrin selenium (ITS), and dexamethasone, all sourced from Sigma-Aldrich (USA). TGF-β3 was acquired from Miltenyi Biotec (Germany), while MTT was obtained from Kiazist (Iran). The PRP extraction kit was supplied by Rooyagen (Iran), and GO nanopowders with a purity of 99% were sourced from US Research Nanomaterials (USA). Additional materials, such as Alg, Gel type B, fluorescein diacetate, propidium iodide, Alcian Blue, glutaraldehyde, and calcium chloride, were also obtained from Sigma-Aldrich (USA). FSP SYBR Green qPCR Mix 2X, TRIZOL reagent, and cDNA synthesis kits were purchased from Fanavari Salamat Partogene (Iran).

### Bioink preparation

The graded OC scaffold was fabricated using two distinct bioinks composed of Alg, Gel, GO and Alg, Gel, PRP. Three distinct phases were developed: one phase containing optimal Alg-Gel-GO bioink for the subchondral bone, another consisting of calcified cartilage with Alg-Gel-varying gradient of PRP and the optimal Alg-Gel-GO bioink, and an articular cartilage phase made of Alg-Gel- varying gradient of PRP bioink. To prepare a uniform solution, 10% Alg and 6% Gel were dissolved in phosphate-buffered saline (PBS). The printing ink for the bone phase was created by adding GO nanopowders to the Alg and Gel mixture at various concentrations (0%, 1%, and 2% w/v). Additionally, bioinks for the cartilage phase were formulated by mixing Alg-Gel with varying lyophilized-PRP concentrations (0%, 1%, 2%, and 3% w/v). Table 1 provides an overview of the experimental groups, and each group will be referred to by the abbreviations listed.

**Table 1:** The bioink types and their abbreviations for each phase.

Tissue phase	Bioink name	Alg (% w/v)	Gel (% w/v)	GO (% w/v)	PRP (% w/v)
Subchondral bone	Alg-Gel-GO_0_	10	6	0	0
Subchondral bone	Alg-Gel-GO_1_	10	6	1	0
Subchondral bone	Alg-Gel-GO_2_	10	6	2	0
Calcified cartilage	Alg-Gel-GO_1_-PRP_0_	10	6	(Optimal dosage)	0
Calcified cartilage	Alg-Gel-GO_1_-PRP_1_	10	6	(Optimal dosage)	1
Calcified cartilage	Alg-Gel-GO_1_-PRP_2_	10	6	(Optimal dosage)	2
Calcified cartilage	Alg-Gel-GO_1_-PRP_3_	10	6	(Optimal dosage)	3
Articular cartilage	Alg-Gel-PRP_0_	10	6	0	0
Articular cartilage	Alg-Gel-PRP_1_	10	6	0	1
Articular cartilage	Alg-Gel-PRP_2_	10	6	0	2
Articular cartilage	Alg-Gel-PRP_3_	10	6	0	3

### Triphasic scaffolds fabrication and characterization

3D-printed scaffolds were created in Solidworks software and converted to G-code using Simplifier. Lattice structures were fabricated using a BioFabX4 bioprinter with bioink extruded through a 500-µm nozzle at 0.6 atm pneumatic pressure. Each layer consists of parallel struts 90° to the layer below. The printing rate was maintained at 3 mm/s, ensuring that struts with a width of approximately 1.6 mm were deposited. The structure designed for printing featured a grid layout with overall dimensions of 9.8 × 9.8 × 1.2 mm^3^, consisting of four layers of 1.2 mm each designated for bone and cartilage an interfacial phase among them, totaling 12 layers for the triphasic scaffold. Following printing, the scaffolds were immediately soaked in a crosslinking solution including 2% w/v CaCl_2_ and kept at room temperature for 15 min to preserve their shape and structural stability. To obtain the best results, the printing parameters were changed for each group of triphasic scaffolds. Table 2 lists the names of the various 3D-printed triphasic scaffold groups. A constant 1% (w/v) GO solution was used for triphasic scaffold preparation following subchondral layer optimization.

**Table 2:** Abbreviations for the names of 3D-printed triphasic scaffold groups and their compositions at each phase.

Scaffold group names	Subchondral bone layer composition	Calcified cartilage layer composition	Articular cartilage layer composition
Alg-Gel-GO-PRP_0_	Alg-Gel-GO_1_	Alg-Gel-GO_1_-PRP_0_	Alg-Gel-PRP_0_
Alg-Gel-GO-PRP_1_	Alg-Gel-GO_1_	Alg-Gel-GO_1_-PRP_1_	Alg-Gel-PRP_1_
Alg-Gel-GO-PRP_2_	Alg-Gel-GO_1_	Alg-Gel-GO_1_-PRP_2_	Alg-Gel-PRP_2_
Alg-Gel-GO-PRP_3_	Alg-Gel-GO_1_	Alg-Gel-GO_1_-PRP_3_	Alg-Gel-PRP_3_

### Subchondral bone layer characterization

#### Swelling ratio

To estimate the swelling ratio of scaffolds, we initially measured the dry weight of 3D-printed Alg-Gel-GO_0_, Alg-Gel-GO_1_, and Alg-Gel-GO_2_ scaffolds (*n* = 3) before immersing them in the PBS solution. The scaffolds were removed, dried, and then weighed at different times to calculate their initial moisture content, otherwise known as their wet weight. The percentage of swelling ratio was calculated using Eq. (1), where m_D_ and m_S_ represent the masses of scaffolds before and after swelling in PBS, respectively.

1 $$\:Swelling\:ratio=\frac{{m}_{S}{-m}_{D}}{{m}_{D}}\times\:100\%$$

#### Mechanical properties

The mechanical behavior of Alg-Gel scaffolds containing GO were analyzed using an uniaxial compression test on a SANTAM STM-20 universal testing machine (Iran). Cylindrical specimens were fabricated, immersed in CaCl_2_, washed with deionized water, and then freeze-dried. A compressive force of 5 kN was applied at a crosshead rate of 1 mm/min to five specimens until they experienced a 60% decrease in height. The compressive strength was determined using Eq. (2), where F denotes the applied maximum force and A represents the specimen’s cross-sectional area.

2 $$\:S=\frac{F}{A}$$

#### Fourier transform infrared spectroscopy (FTIR)

The chemical properties of the Alg-Gel scaffold prepared with different dosages of GO (0%, 1%, and 2% w/v) were assessed using FTIR (Thermo Nicolet Avatar 360, USA) in the range of 400–4000 cm^− 1^. The analysis was performed under a pressure of 250 Pa, with a resolution of 4 cm^− 1^, and it involved averaging 100 scans for enhanced precision.

#### Rheological properties

The rheological properties of Alg-Gel bioinks with (0%, 1%, and 2% w/v) GO were evaluated using an Anton-Paar Physica MCR300 rheometer with a 25 mm plate-plate geometry. The assessment involved frequency sweep tests operated at 0.1 to 100 Hz with a strain of 1.0% and steady-state flow measurements to analyze viscosity against shear rate from 0.01 to 1000 s⁻¹. Viscosity recovery was examined by applying sequential shear rates of 0.1 s⁻¹ for 60 s, followed by 100 s⁻¹ for 10 s, then returning to 0.1 s⁻¹ for another 60 s. This sequence simulates bioink behavior before, during, and after printing. All tests were completed at 25 °C.

#### Cell viability assay

The biocompatibility of Alg-Gel bioinks with GO (0%, 1%, and 2% w/v) was evaluated via MTT assay using L929 cells (Pasteur Institute of Iran). Cells (5 × 10^3^ per well) were seeded on sterilized hydrogels in 96-well plates^15^, and MTT assays were performed after 1, 3, and 7 days. After removing the culture media, 10 µL MTT reagent and 100 µL serum-free media were added to each well, followed by a 4 h incubation at 37 °C (5% CO_2_, 95% humidity) in the dark. After incubation, the media was removed, 100 µL of solubilizing solution was added, and the plate was shaken in the dark for 15–20 min to dissolve the MTT formazan. Absorbance was determined at 570 nm utilizing a microplate reader (Bio-Rad Laboratories). The cell viability percentage was analyzed using Eq. (3), with a 2D cell culture medium as the reference.

3 $$\:Cell\:viability\%=\frac{{Mean\:OD}_{sample}}{{Mean\:OD}_{control}}\times\:100$$

In addition, a field-emission scanning electron microscope (FE-SEM, MIRAIII, TESCAN, Czech Republic) was used to observe the cell morphology seeded on the scaffolds. L929 cells were seeded on scaffolds in each group at a density of 8 × 10^3^ cells/well. After 48 h of incubation, the cells were washed with PBS, fixed with 2.5% (v/v) glutaraldehyde, and then dehydrated using varying concentrations of ethanol (50%, 70%, 90%, 95%, and 100%) for 5 min each. Eventually, the L929 cell-attached scaffolds were coated with gold-palladium and viewed with FE-SEM.

### Lyophilized PRP powder preparation and characterization

To obtain PRP, 40 mL of human peripheral blood was collected from healthy volunteers, and 5 mL of acid citrate dextrose solution-A was added as an anticoagulant. A 1 mL blood sample was subjected to a complete blood count. The remaining blood was centrifuged at 1600 rpm for 15 min to create three layers: red blood cells at the bottom, leukocytes in the middle, and plasma on top. The buffy coat and plasma were centrifuged at 2800 rpm for 7 min to concentrate platelets, resulting in 4–6 mL of leukocyte-rich PRP. Platelet counts in the PRP were determined using a hematology analyzer (Sysmex XN 1,000), averaging 540 × 10^3^ platelets/µL. The PRP was manually homogenized by inversion, resuspended in 100 mM trehalose, frozen at −80 °C, and freeze-dried at −40 °C for 24 h. The final PRP powder was stored at −20 °C for future use. The isolated PRP was characterized by enzyme-linked immunosorbent assay (ELISA) and FTIR^16^. The in vitro release analysis measured the GFs released from PRP using an ELISA method with specific kits for PDGF-B (R&D Systems, cat. No. DBB00) and TGF-β1 (R&D Systems, cat. No. DY240). The ELISA procedure adhered to the manufacturer’s guidelines, which included incubation with assay diluent, addition of control samples, washing, and application of detection antibodies, with optical density measured at 450 nm with a microplate reader. FTIR analysis of the lyophilized PRP powder was conducted over a range of 400–4000 cm^− 1^, at a pressure of 250 Pa, using a spectral resolution of 4 cm^− 1^ and 100 scans.

### Isolation, expansion, and confirmation of rat BM-MSCs

Using the following technique, BM-MSCs were isolated from the tibiae and femurs of rats^17^. The epiphyses were removed, and the bone marrow was flushed out using DMEM/F12 medium supplemented with 10% FBS, 100 U/mL streptomycin, and 100 µg/mL penicillin. The medium was changed after the cells were cultivated for 2 days at 37 °C in an atmosphere containing 5% CO_2_ to eliminate non-adherent cells. Once the cells reached 90% confluence, BM-MSCs were utilized for subsequent in vitro tests at passage 3. Flow cytometry was employed to confirm the presence of specific cell surface antigen markers in rat BM-MSCs. Antibodies against positive markers (CD73, CD105, and CD90) and negative markers (CD45 and CD34) were utilized.

### 3D-printed triphasic scaffold characterizations

#### Printability assessment of bioinks

Shape fidelity was evaluated through qualitative analysis of extruded filaments using macroscopic images, along with the determination of the printability index (Pr) based on the method defined by Ouyang et al.^18^. To assess printability, 500 μm nozzles were installed on 10 mL plastic syringes, each filled with 5 mL of a hydrogel blend for testing. We established the minimum pressure required for consistent extrusion while adjusting the nozzle speed and bioink temperature to optimize printing precision and duration. The infill density was set to 100% to prevent unwanted porosity in the constructs, and the distance between the needle and the printing surface was modified for calibration purposes. To improve visualization, we mixed two drops of food dye into the bioinks. The printability index (Pr) was then measured with normalizing the pore perimeter to the pore area, as described in Eq. (4).

4 $$\:Pr=\frac{{L}^{2}}{16A}$$

The perimeter is represented by L, and the area of the enclosed grid hole is denoted by A. Under optimal gelation conditions or ideal printability, the interconnected channels of the constructs would form a perfect square, resulting in a Pr value of 1. A higher Pr value indicates a greater degree of bioink gelation, while a lower Pr value signifies a lesser degree of gelation. The Pr value for each set of printing parameters was calculated by analyzing optical images of the printed constructs. The perimeter and area of the interconnected channels were measured using ImageJ software (*n* = 5).

#### Morphological characterization

The surface characteristics and pore arrangement of the manufactured scaffolds were examined and documented using FE-SEM. The cross-sections of the freeze-dried specimens were affixed to aluminum foil and subsequently coated with gold. The diameters of the struts and pores were measured using ImageJ software.

#### Physical characterization

The degradation behavior of triphasic scaffolds with varying percentages of PRP (0%, 1%, 2%, and 3% w/v) was assessed by measuring the weight loss in the PBS at 37 °C over a period of 28 days. After determining the initial dry weight (m_0_), the scaffolds were immersed in the PBS solution. The dry weight of the specimens was recorded at 7, 14, 21, and 28 days to calculate the weight loss of the scaffolds (m_1_). The degradation rate (D) of each scaffold was computed using Eq. (5):

5 $$\:D=\frac{{m}_{0}{-m}_{1}}{{m}_{0}}\:\times\:100\%$$

#### Mechanical and rheological characterizations

The compressive strength of triphasic scaffolds was determined as described in Sect. 2.4.2. The rheological properties of the triphasic scaffolds containing varying percentages of PRP (0%, 1%, 2%, and 3% w/v) were evaluated with a rheometer equipped with a plate-plate geometry (25 mm diameter) at 25 °C. A frequency sweep was conducted from 0.1 to 100 Hz with a 1% strain amplitude to determine the storage modulus (G’) and loss modulus (G″) as functions of frequency. The viscosity of the bioinks was measured at a consistent shear rate of 100 s^− 1^ to the power of minus one over the entire duration of 200 s.

#### Biocompatibility characterization of triphasic scaffold

To evaluate the compatibility and cell proliferation of Alg-Gel scaffolds with varying concentrations of PRP (0%, 1%, 2%, and 3% w/v) and the optimal dosage of GO, an MTT assay was conducted. The assay involved seeding BM-MSCs within sterilized hydrogels in 24-well plates at a density of 10 × 10^3^ cells per well. After 1, 3, and 7 days, the MTT kit was utilized as described in Sect. 2.4.5. Cell viability on the 3D-printed scaffolds was assessed through a live/dead assay. BM-MSCs were seeded on the triphasic scaffolds and cultured in a growth medium. Cell viability was evaluated after 72 h, using the fluorescein diacetate (FDA) and propidium iodide (PI)^19^. The procedure involved removing the media and rinsing the scaffolds twice with PBS (1X). Subsequently, the staining solution (PI at 2 mg/mL, FDA at 5 mg/mL, and culture medium without FBS) was applied to the cells, then was incubated and the staining solution was removed after 10 min. An Olympus IX70 fluorescent microscope was used to observe living cells, which were stained fluorescent green by the FDA, and dead cells, which were stained red with PI in the hydrogel. The morphology of the cells seeded on the scaffolds was analyzed using FE-SEM. BM-MSCs were seeded on the scaffolds in each group at a density of 3 × 10^3^ cells/well. After 48 h of incubation, the stem cells were washed with PBS, fixed with 2.5% (v/v) glutaraldehyde, and dehydrated using a series of ethanol concentrations (50%, 70%, 90%, 95%, and 100%) for 5 min each. Finally, the scaffolds with attached stem cells were coated with gold-palladium and examined using FE-SEM.

### In vitro chondrogenesis assay

At passage 3, a BM-MSCs suspension with a cell concentration of 3 × 10^5^ cells/mL was seeded on the triphasic scaffold groups in a culture medium (DMEM/F12 supplemented with 10% FBS and 1% penicillin-streptomycin) and incubated for 4 h at 37 °C in a 5% CO_2_ atmosphere to promote initial adhesion. The control group was prepared by placing the same number of cells in wells without scaffolds. Subsequently, the BM-MSCs seeded on the triphasic scaffold groups were incubated in a chondrogenic differentiation medium. The chondrogenic differentiation medium, which consisted of DMEM with a D-glucose concentration of 4.5 g/L supplemented with P/S, 120 µM ascorbic acid 2-phosphate, 40 µg/mL L-proline, 10^− 7^ M dexamethasone, ITS + 1 (insulin-transferrin-sodium selenite, linoleic acid-BSA), and 10 ng/mL TGF-β3, was replaced three times per week^20^.

#### Gene expression analysis

After 3 weeks of incubation in a chondrogenic differentiation medium, total RNA was extracted using TRIzol-Chloroform. The mixture was then centrifuged at 12,000 rpm for 15 min, and the temperature was kept at 4 °C following the addition of chloroform. The samples were stored at −20 °C overnight. The next day, RNA was precipitated and analyzed for quality with a NanoDrop spectrophotometer (Thermo Fisher Scientific, USA). A cDNA synthesis kit was used for complementary DNA (cDNA) synthesis, followed by incubation at 25 °C for 10 min, 47 °C for 60 min, and 85 °C for 5 min in a thermocycler. Reverse transcription-polymerase chain reaction (RT-PCR) was applied using SYBR Premix Ex Taq II master mix (TaKaRa) in a Rotor-Gene Q MDx (USA). Chondrogenic differentiation-related genetic markers, such as collagen II, collagen I, and SOX9 were evaluated. The reference gene Glyceraldehyde-3-phosphate dehydrogenase (GAPDH) was employed to normalize the target genes, following the procedure outlined by Lotfi et al.^21^. The resulting cycle threshold (CT) was converted into 2^−ΔΔCT^, and the necessary analysis were performed. The results are reported as relative expression (fold change). The primers used for RT-PCR and their information are listed in Table 3.

**Table 3:** The primers used in RT-PCR and their information.

Gene name	Accession number	Sequence	Product size (bp)	T_m_ (℃)
Collagen type II	NM_001414896.1	F: 5´-ATCTGTGAAGACCCAGACTGC-3´	121	60
		R: 5´-GTTCTCCTTTCTGCCCCTTTGG-3´		
Sox9	NM_080403.3	F: 5´-AGTCGGTGAAGAATGGGCAA-3´	158	60
		R: 5´-CTGAGATTGCCCGGAGTGC-3´		
Collagen type I	NM_053304.1	F: 5´-GTACATCAGCCCAAACCCCA-3´	87	60
		R: 5´-TCGCTTCCATACTCGAACTGG-3´		
GAPDH	NM_017008.4	F: 5´-TGTTCTAGAGACAGCCGCAT-3´	93	60
		R: 5´-CGATACGGCCAAATCCGTT-3´		

#### Histological analysis

On day 21, Alcian Blue staining was employed to detect the presence of glycosaminoglycans (GAGs) deposits on the triphasic constructs. At predetermined time intervals, the 3D-printed constructs with BM-MSCs were treated with Alcian Blue solution for staining^22^. The scaffolds were washed with PBS, fixed in 10% formalin for 30 min, and then rinsed. Each scaffold with differentiated cells was incubated with 1% Alcian Blue solution (in 3% acetic acid, pH 2.5) for 30 min. After staining, the excess dye was removed, and the scaffolds were rinsed thoroughly. Images were taken with a Zeiss microscope (Germany).

#### Cumulative growth factor release

The scaffold (Alg-Gel-Go-PRP2) was established to examine the release of growth factors (TGF-β, PDGF) from hydrogels. In vitro release studies were performed in a CO2 incubator at 37 °C over a duration of 21 days. Hydrogels were deposited into 24-well plates, subsequently supplemented with 1 mL of DMEM. At designated intervals, 50% of the supernatant was extracted, and the medium was replenished. The obtained supernatants were preserved at −80 °C until analysis. Commercially accessible ELISA kits (Thermo Fisher, USA) were utilized to assess the secretion of PDGF and TGF-β1 in accordance with the manufacturer’s instructions, with absorbance measured at 420 nm. The release kinetics and mechanism were determined from data obtained from the in vitro cumulative release graph. 60% of the drug release data were utilized for the Korsmeyer-Peppas model.

### Statistical analysis

The data are presented as means ± standard deviations from three measurements (*n* = 3). Statistical analysis was conducted using one-way or two-way ANOVA, using GraphPad Prism software. A significance threshold of *P* < 0.05 was used, with * (*P* < 0.05), ** (*P* < 0.01), and *** (*P* < 0.001) indicating varying levels of significance.

### Ethical approval statement

The investigation obtained the necessary clearance from the research ethics committee of the Iran University of Medical Sciences in Tehran, Iran, which has the Ethical Code IR.IUMS.REC.1401.978. All experiments adhered to relevant guidelines and regulations, which were endorsed by the Research Ethics Committee of the Iran University of Medical Sciences, located in Tehran, Iran.

#### Human samples (PRP extraction)

All blood samples used for PRP extraction were obtained with written informed consent from all donors. The study protocol was approved by the Research Ethics Committee of the Iran University of Medical Sciences, Tehran, Iran (Ethical Code IR.IUMS.REC.1401.978), and all procedures were conducted in accordance with relevant guidelines and regulations.

#### Animal experiments (rat BM-MSCs isolation)

All experimental protocols involving rats were approved by the Research Ethics Committee of the Iran University of Medical Sciences, Tehran, Iran (Ethical Code IR.IUMS.REC.1401.978). All methods were performed in accordance with relevant guidelines and regulations and reported following the ARRIVE guidelines. Three-week-old male specific pathogen-free Wistar rats were sourced from the Experimental Studies Center at Iran University of Medical Sciences. Ketamine (60 mg/kg) and xylazine (8 mg/kg) were used to anesthetize the rats prior to euthanasia via carbon dioxide (CO_2_) inhalation.

## Results and discussion

### Physicochemical characterization of the subchondral bone layer

#### Swelling ratio analysis

**Fig. 2:**
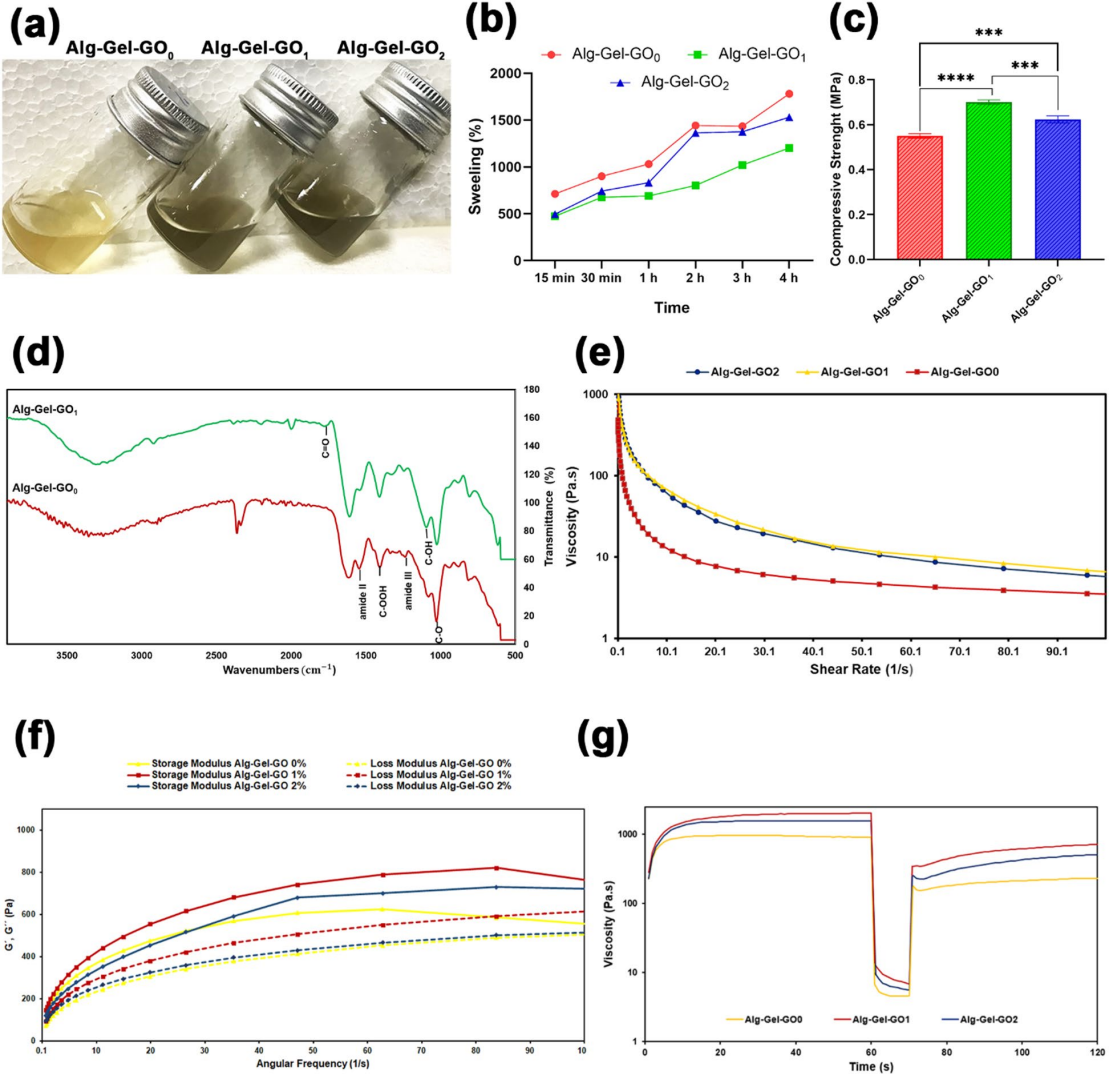
(**a**) Image of the subchondral layer bioinks with different GO concentrations. (**b**) Swelling ratio in PBS as a function of immersion time of scaffolds with various GO concentrations. (**c**) Compressive strength of bioinks with different GO content; (ns, no significant difference; ^*^p ≤ 0.05, *** p ≤ 0.001, **** p ≤ 0.0001). (**d**) FTIR spectra of Alg-Gel-GO_0_ and Alg-Gel-GO_1_ bioinks. Rheological properties of GO-containing hydrogels: (**e**) Viscosities versus different shear rates, (**f**) Dependence of storage modulus (G’) and loss modulus (G”) on different angular frequencies, and (**g**) Recovery behavior.

Bioinks of the subchondral bone layer with different GO concentrations (0%, 1%, and 2% w/v) were prepared as explained previously **(**Fig. 2a**)**. The ability of a material to absorb water is a key factor to consider, because cells take in nutrients from the surrounding environment to sustain their mobility, growth, and proliferation. This is also related to the functioning of scaffold structures and the precise spatial distribution of cells^23,24^. According to the results **(**Fig. 2b**)**, the Alg-Gel-GO_0_ scaffold exhibited a notably greater swelling ratio than the Alg-Gel-GO_1_ and Alg-Gel-GO_2_ scaffolds.

The GO-free scaffold exhibited 1700% swelling in PBS, making it difficult to handle because of its weak mechanical behaviors. In GO-containing scaffolds, the swelling ratio after 4 h of immersion was lower than that of the GO-free scaffolds, with values of 1200% and 1530% for Alg-Gel-GO_1_ and Alg-Gel-GO_2_, respectively. As GO is a hydrophobic material, those containing GO exhibit lower water absorption capacity as those without GO. Similar findings regarding the swelling ratio with GO addition were reported by Hu et al.^25^. Water uptake in Alg-Gel-GO_2_ increased as the GO content increased after 4 h. The mechanical and structural stability of Alg-based printed scaffolds is critical. Divalent ions, or ionic crosslinkers, can separate from ionically cross-linked Alg hydrogels, decrosslinking the scaffolds and resulting in a loss of structural stability^26^. According to the findings, incorporating GO into Alg helps maintain the structural integrity of Alg-based scaffolds. Good interfacial adhesion between GO and Alg chains, along with hydrogen bonding, is likely to lead to higher scaffold stability. In an investigation by Choe et al.^26^, a lack of Ca^2+^ ions caused swelling in Alg scaffolds with an Alg content of 3%; at the same time, GO into Alg decreased the swelling ratio and improved the structural stability of Alg-based scaffolds within 5 days of incubation. A higher GO content in the Alg-Gel-GO_2_ scaffold caused more water molecules and hydrophilic protein uptake, leading to a higher swelling ratio. Consequently, the interaction between GO and Alg polymer chains enhances the mechanical integrity of the printed scaffolds at a GO content of approximately 2.0 mg/mL, thereby improving structural stability.

#### Mechanical property analysis

The mechanical strength, a crucial aspect in the fabrication of scaffolds for bone TE, indicates the load-bearing capacity of the materials^27^. According to the results (Fig. 2c), the addition of GO obviously improved the compressive strength (0.72 MPa for 1 wt% and 0.63 MPa for 2 wt% GO) compared to ink without GO (0.55 MPa). The 3D-printed scaffolds with Alg-Gel-GO_0_ exhibited brittleness, making them difficult to handle, with edge sections detaching due to weak mechanical integrity. According to the results, incorporating GO into Alg can improve scaffolds’ structural stability. The increase in mechanical behavior as the GO content increases is attributed to the further formation of bridges through GO nanofillers, which reinforces the 3D structure of the scaffolds^10^. On the other hand, interactions such as hydrogen bonding among the polymer matrix (Alg chains) and the GO nanosheets, due to their good compatibility, increased the compressive strength^24^. The improved strength of the GO-incorporated inks compared with the inks without GO highlights the reinforcement effect of GO incorporation. As shown in Fig. 2c, the scaffold with 1 wt% GO exhibits a higher compressive strength than that with 2 wt% GO, revealing a non-linear relationship between enhancing the mechanical properties of the scaffold and an enhancement in the GO concentration. When the incorporation of GO is excessively high, the structure is susceptible to collapse, likely due to the swelling and high-water absorption capacity of GO, which reduces the structural stiffness. The compressive stiffness is closely linked to water content; with increase in the water content, the structure becomes more brittle and permeable. An optimal amount of GO should be added to obtain appropriate mechanical properties of the scaffold.

#### FTIR analysis

The FTIR spectra of the Alg-Gel-GO_0_ and Alg-Gel-GO_1_ hydrogels are shown in Fig. 2d. Since the FTIR spectra of Alg-Gel-GO_1_ and Alg-Gel-GO_2_ do not display differences between the peaks, the spectra of Alg-Gel-GO_2_ are not shown in the FTIR figure. The band at 1027 cm^− 1^ represents a characteristic peak for Alg, confirming its glucuronic acid unit^28^. The peak associated with the carboxyl groups present in the Alg is observed at 1409 cm^− 1^^29^. A prominent absorption peak occurs at 1614 cm^− 1^, which is associated with the asymmetric stretching vibrations of the -COO⁻ group^30^. For Gel, the characteristic peaks at 1544 and 1237 cm^− 1^ are attributed to Amide II and Amide III, respectively^29^. These characteristic peaks were also observed in the Alg-Gel-GO_1_ spectra. The FTIR analysis revealed a characteristic GO peak at 1777 cm^− 1^ in the Alg-Gel-GO_1_ spectra. The region spanning 1700 to 2200 cm^− 1^ is related to the C = O stretching motions of the − COOH group, which can be ascribed to C = O linkages found within carbonyl components^29–31^. The peak around 1095 cm^− 1^ represents the C–OH bending mode of GO^31^. Furthermore, the interaction between GO and Alg-Gel hydrogel led to the broadening of the peak occurring at 2900–3600 cm^− 1^^11^.

#### Rheological analysis

Proper viscosity is crucial for successful printing, and assessing the rheological behavior of biomaterials can help identify the optimal printing characteristics^32^. The viscosity-shear rate curves of bioink formulations with various GO contents are shown in Fig. 2e. The GO-containing bioinks exhibited higher viscosities than the GO-free bioink (Alg-Gel-GO_0_). Incorporating GO into the Alg-Gel bioink increased its viscosity and enhanced its shear-thinning behavior, making it applicable for 3D printing. A similar finding was reported by Zhang et al.^24^, who observed that viscosity increased with GO concentration in the Alg-Gel bioink. A common characteristic observed across all tested bioinks was their shear-thinning behavior, with a consistent decrease in viscosity as the shear rate was raised. This shear-thinning property is vital for bioinks to be effectively extruded from the nozzle at low pressure while preserving the printed structure’s shape and dimensions post-printing^26^. The bioink viscosities decreased with higher GO concentrations, with measurements at a shear rate of 1 s^− 1^ showing viscosities of 92, 379, and 376 for Alg-Gel-GO_0_, Alg-Gel-GO_1_, and Alg-Gel-GO_2_, respectively. Frequency sweep tests revealed that all bioinks presented a larger storage modulus (G’) than loss modulus (G’’), representing solid-like behavior and suitability for printing (Fig. 2f). The addition of GO to the Alg-Gel bioink improved the storage modulus, suggesting enhanced structural integrity. GO incorporation also increased both the storage and loss moduli, indicating improved viscoelastic properties, printability, and shape fidelity, as the interactions between Alg and GO resulted in a more viscous and stable hydrogel. Increasing the GO concentration to 2% resulted in a reduction of both moduli, with the highest modulus recorded at a concentration of 1%. The thixotropic behavior of hydrogels is a key factor in the printability and resolution of 3D-printed structures^10^. Figure 2g shows the viscosity recovery behavior of Alg-Gel-GO bioinks. For Alg-Gel-GO_0_, the initial viscosity was 595 Pa.s. Upon increasing the shear rate to 100 s^− 1^ (Step I), the viscosity sharply decreased to 4.54 Pa.s. With elimination of the shear rate (Step II), the viscosity recovered to 236 Pa.s within 60 s, reaching 39.7% of the initial value. In contrast, the Alg-Gel-GO_1_ and Alg-Gel-GO_2_ bioinks recovered 98.54% and 82.37% of their initial viscosity, respectively. Both bioinks exhibited a smaller viscosity decrease in Step II compared to Alg-Gel-GO_0_, although this did not significantly affect their printability because of the similar viscosities. The high viscosity of Alg-Gel-GO0 reduced its recovery value. The incorporation of GO significantly improved viscosity recovery, thereby enhancing the viscosity and stability. These rheological properties ensure the layer-by-layer integrity of 3D-printed scaffolds, preventing collapse or destruction during printing.

### Biocompatibility analysis of subchondral bone layer

**Fig. 3:**
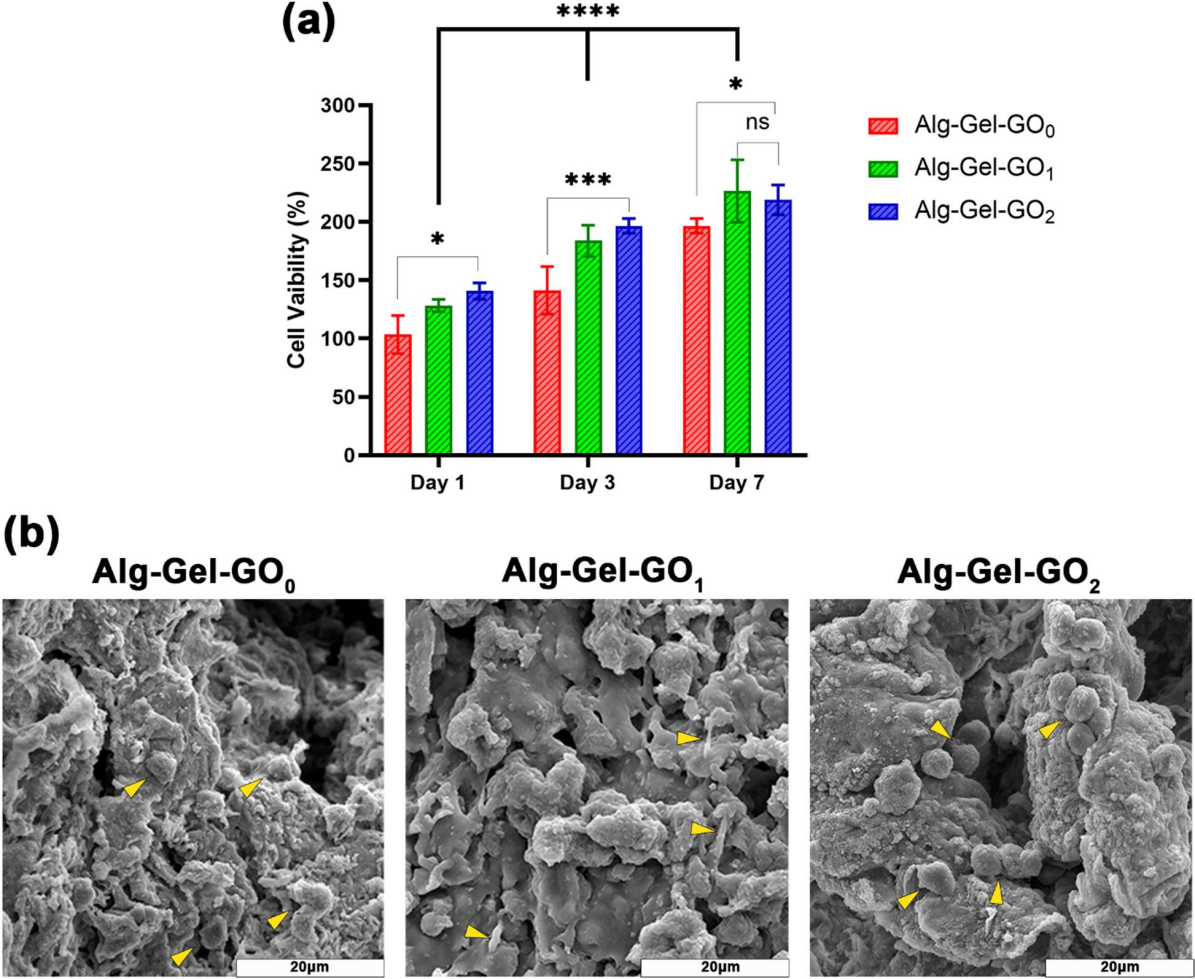
Evaluation of seeded cell viability on scaffolds. (**a**) Cell viability across different concentrations of GO was compared using the MTT assay; Data expressed as mean ± SD (*n* = 3); ns: no significant difference, *p < 0.05, **p < 0.01, *** *p* < 0.001, **** *p* ≤ 0.0001. (**b**) FE-SEM images of the adhesion of L929 cells on the surface of Alg-Gel-GO_0_, Alg-Gel-GO_1_, and Alg-Gel-GO_2_.

The effect of GO concentration on cell viability and morphology were evaluated using MTT and FE-SEM, respectively. In Fig. 3a, the MTT results indicate L929 fibroblast cell viability in hydrogels on days 1, 3, and 7. The results indicate that all Gel-Alg-GO groups exhibited non-cytotoxic effects over 7 days. Cell viability was observed across all groups, with increased concentrations of GO resulting in a decrease in cell death, showing significant variations between the Alg-Gel-GO_1_ and Alg-Gel-GO_2_ groups compared to the Alg-Gel-GO_0_ group on days 1 and 7. This finding is consistent with other studies reporting no adverse effects from scaffolds containing GO. Similarly, researchers observed no cytotoxicity at low GO concentrations (1–3 µg/mL)^33,34^. However, higher concentrations (≥ 100 µg/mL) have been shown to induce cytotoxicity, as demonstrated by Lim et al.^35^. Furthermore, the data indicated that the cell proliferation rate was considerably higher over time in all Alg-Gel-GO groups, suggesting that these scaffolds effectively support cell proliferation. However, after 7 days, a slight decrease in cell viability was perceived in the Gel-Alg-GO_2_ group in comparison to the Gel-Alg-GO_1_group. Our findings are similar to those of Zhang et al.^24^, who developed a novel MSC-laden Alg-Gel-GO composite bioink and noted improved cell viability with higher GO concentrations. Higher GO concentrations (1% and 2% GO w/v) supported earlier cell spread by day 7 compared with 0.5% and 0% GO. However, cell proliferation decreased at the 2% GO concentration over time. The attachment of L929 fibroblast cells to the scaffolds was analyzed using FE-SEM (Fig. 3b). The cells displayed good spread and elongation on the subchondral layer scaffolds, especially with a 1% increase in GO concentration. The morphological analysis results align with the MTT assay findings and previously reported literature^36,37^. This enhanced cell attachment is likely due to the presence of negatively charged carboxylic, hydroxyl, and epoxy groups in the Alg-Gel-GO nanocomposite scaffolds^38^. Based on the MTT and cell attachment test results, the Alg-Gel-GO_1_ group seems to be a promising candidate for enhancing cell proliferation and adhesion.

### Lyophilized PRP powder characterizations

**Fig. 4:**
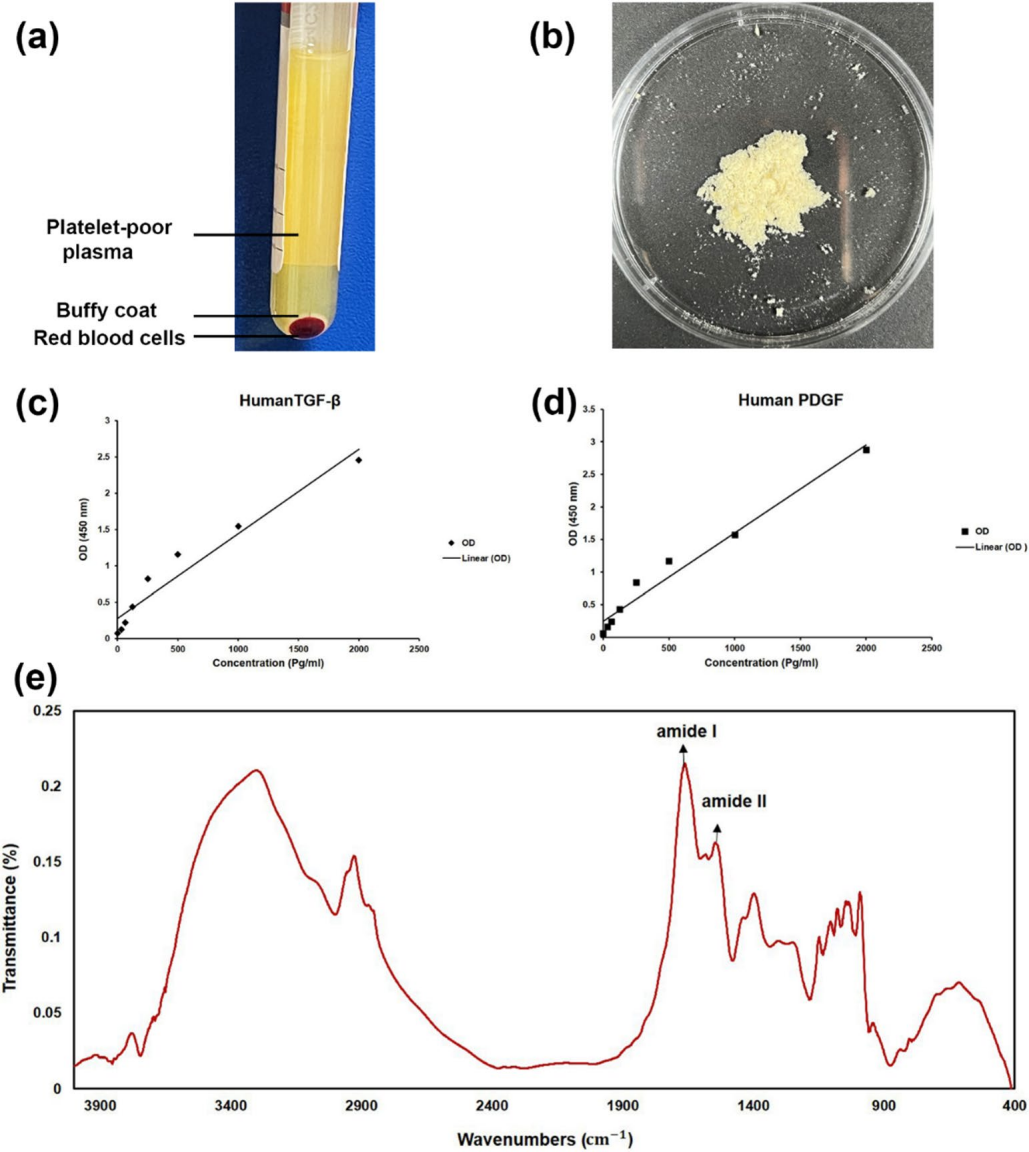
(**a**) Isolated PRP from human peripheral blood. (**b**) Lyophilized PRP powder. Release of GFs, including (**c**) PDGF and (**d**) TGF-β1 from PRP. (**e**) FTIR spectra of PRP powder.

Figure 4a shows the isolated PRP and Fig. 4b shows the lyophilized PRP powder. The kinetics of TGF-β1 and PDGF release from the lyophilized PRP powder were evaluated using ELISA. The findings demonstrated that TGF-β and PDGF concentrations increased with PRP concentration and that GFs were released from PRP roughly linearly (Fig. 4c,d). These factors raise the growth and proliferation of bone and cartilage cells in the OC and facilitate tissue regeneration^39^. The preparation of the lyophilized PRP powder was confirmed by FTIR, as displayed in Fig. 4e. Peak at 1662 cm^− 1^ is related to the amide I band in the PRP powder. Furthermore, the detected peak at 1546 cm^− 1^ corresponded to the amide II functional group with vibration and bending of the v (N–H) bond. The bands around 1250–1500 cm^− 1^ are possibly assigned to the vibrations of amine groups^40,41^.

### Stem cell characterization

**Fig. 5:**
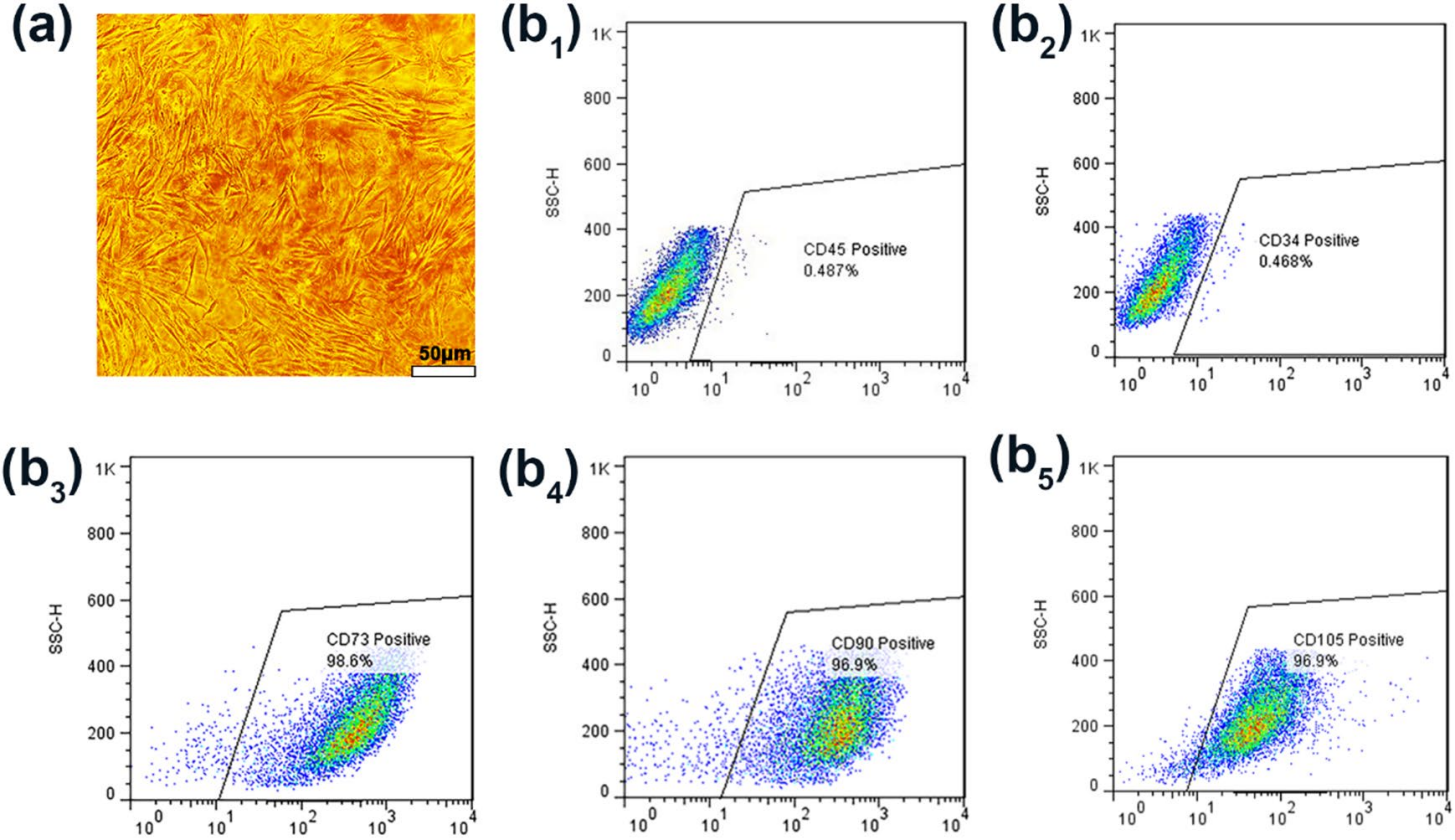
Results of the isolation and characterization of BM-MSCs. (**a**) Morphology of BM-MSCs in passage 3. Scatterplots for the identification of BM-MSCs via flow cytometry. Expressions levels of (**b**_**1**_) CD45, (**b**_**2**_) CD34, (**b**_**3**_) CD73, (**b**_**4**_) CD90, and (**b**_**5**_) CD105 were detected in 0.487%, 0.468%, 98.6%, 96.9%, and 96.9% of the cells, respectively.

BM-MSCs were successfully extracted from the femurs of rats and used for the biological evaluation of triphasic scaffolds. Figure 5a demonstrates a uniform population of cells with a spindle-shaped morphology. The isolated cells were characterized by evaluating the expression of surface antigen markers, including CD73, CD105, CD90, CD45, and CD34 (Fig. 5b_1–5_). Flow cytometry analysis defined that the BM-MSCs displayed high expressions of CD73 (98.6%), CD90 (96.9%), and CD105 (96.9%), but the levels of CD45 (0.487%) and CD34 (0.468%) expression were negative. The collected data align with earlier research and demonstrate that MSCs exhibit a specific array of surface antigen markers (such as CD73, CD90, and CD105) while lacking the surface antigen markers associated with hematopoietic cell lineages (such as CD45 and CD34)^17^.

### 3D-printed triphasic scaffold characterizations

#### Morphological analysis

**Fig. 6:**
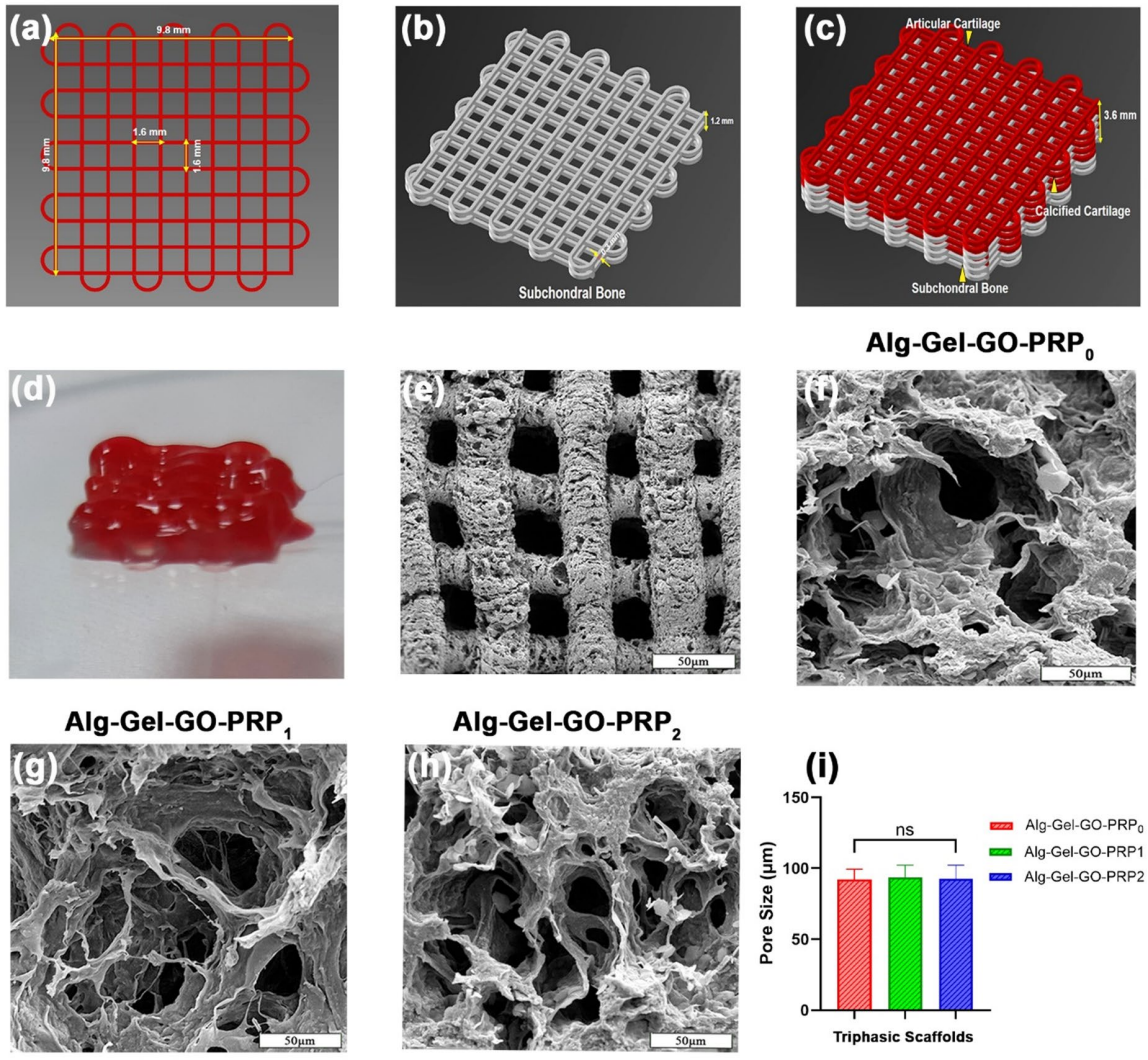
Design and printing of triphasic scaffolds. (**a**) A single-layer structure is designed with a grid arrangement. (**b**) A subchondral bone layer is constructed. (**c**) The printed structures display 12 composed of subchondral bone, calcified cartilage, and articular cartilage. (**d**) A view of the 3D-printed triphasic scaffold is presented. (**e**) FE-SEM images of the printed structure demonstrated the porous grid structure. FE-SEM images of (**f**) Alg-Gel-GO-PRP_0_, (**g**) Alg-Gel-GO-PRP_1_, and (**h**) Alg-Gel-GO-PRP_2_. (**i**) Pore size values of the triphasic scaffolds; (*n* = 3); ns: no significant difference.

Pre-designed CAD models of the 3D-printed scaffolds are displayed in Fig. 6a–c. Using a 3D printer, triphasic scaffolds with a hierarchical structure were successfully fabricated (Fig. 6d). The FE-SEM analysis of the printed structure revealed an open, porous design that facilitates efficient nutrient exchange (Fig. 6e). The structure has an open-space macrogrid layout with interconnecting pores in each grid or 3D-printed filament to facilitate cellular interactions. The highly interconnected porosity of a scaffold supports optimal cell seeding, adhesion, and migration. A hydrogel with high porosity is essential for facilitating the transport of nutrients and oxygen within a 3D construct, especially in the absence of afunctional vascular system^42^. The cross-sectional morphology of lyophilized triphasic scaffold groups, including Alg-Gel-GO-PPR_0_, Alg-Gel-GO-PPR_1_, and Alg-Gel-GO-PPR_2_, was observed by FE-SEM (Fig. 6f–h), and the pore size was determined by analyzing 10 randomly selected FE-SEM images for each sample using ImageJ software. An interconnected porous structure with a characteristic pore size ranging from 10 to 110 μm was observed in all specimens. Since the pores support cellular proliferation and migration, pore size is a crucial property. Previous studies have indicated that a minimum pore size of around 100 μm is essential for OC tissue regeneration because it creates hypoxic conditions that promote chondrogenesis rather than osteogenesis. Figure  6i shows the pore size of the triphasic scaffolds. The pore size of the scaffolds did not change remarkably with increasing the PRP concentration in the Alg-Gel-GO bioink. The 3% PRP group (Alg-Gel-GO-PRP3) could not be reliably printed into a scaffold due to its low viscosity and poor shape fidelity; hence, meaningful data could not be obtained for that group and it was omitted from further analyses.

#### Printability, degradation rate, rheological, and mechanical analysis

**Fig. 7:**
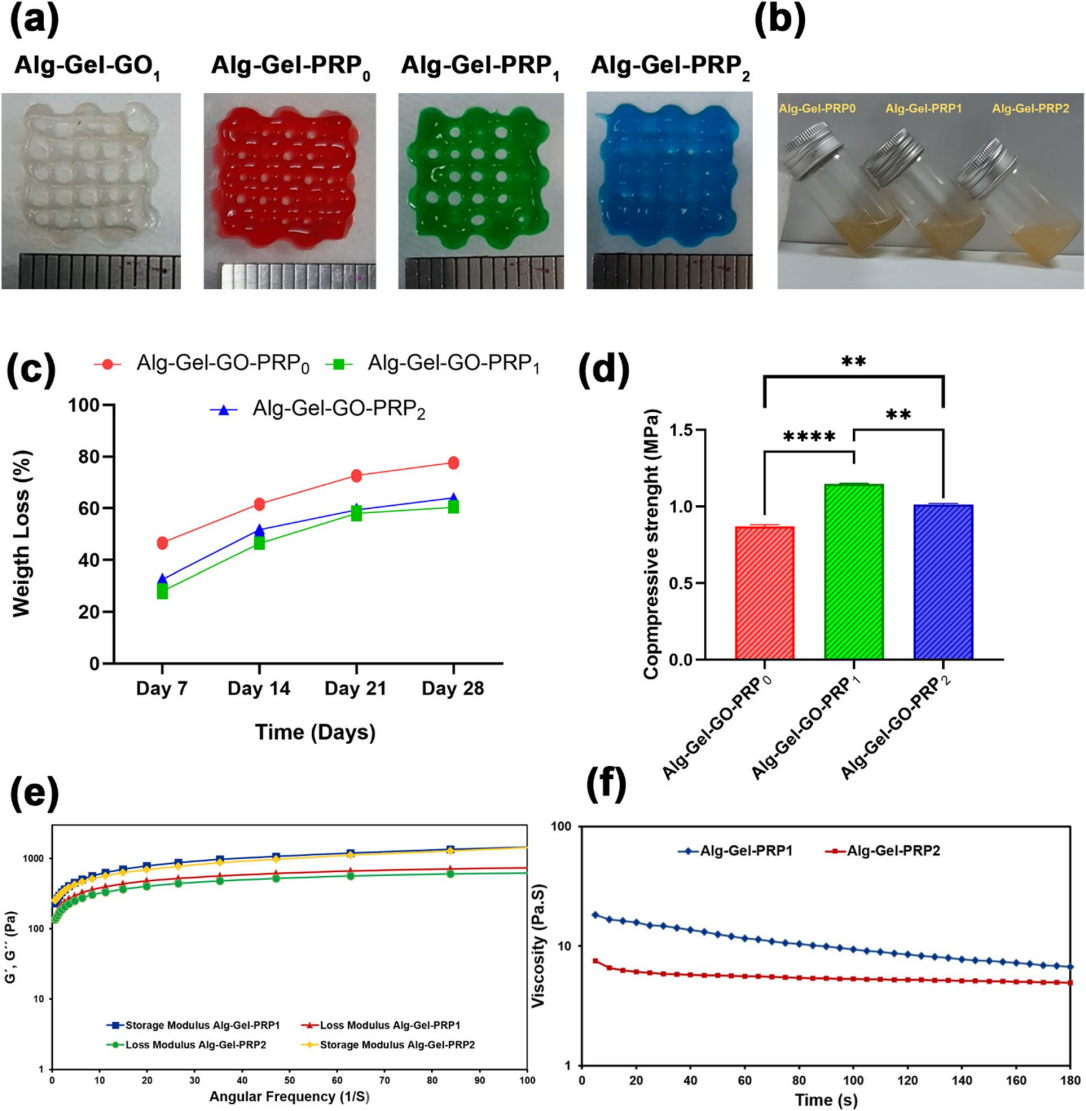
(**a**) Printability assessment of bioink formulation. (**b**) The articular cartilage bioink composition at different PRP concentrations (0%, 1%, and 2%). (**c**) Degradation rate of the 3D-printed triphasic scaffolds within 28 days. (**d**) Compressive strength of the 3D-printed triphasic scaffolds. (**e**) Rheological evaluation of 3D-printed triphasic scaffolds. (**f**) Flow curves of viscosity of 3D-printed triphasic scaffolds. Data expressed as mean ± SD (*n* = 3); ns: no significant difference, *p < 0.05, **p < 0.01, *** *p* < 0.001, **** *p* < 0.0001.

The printability of the bioink was evaluated using techniques such as rheological analysis and the assessment of the integrity of the printed multilayer construct. Figure 7a illustrates the four-layered 3D-printed structure, including Alg-Gel-GO_1_, Alg-Gel-PRP_0_, Alg-Gel-PRP_1_, Alg-Gel-PRP_2_, and Alg-Gel-PRP_3_. However, we encountered some challenges with the Alg-Gel-Go-PRP_3_ sample, because it was not suitable for printing, and we could not create a scaffold with the PRP 3% (w/v) concentration. Therefore, we decided it would be best to exclude this sample from our further characterization efforts. Printing the bioink under optimal gelation conditions produced smooth, uniform filaments that were continuously extruded, forming a well-defined grid structure with distinct, clearly separated layers^18^. To semi-quantitatively assess the printability of the bioink in this study, Pr was calculated for each parameter combination using (Eq. 4). According to Ouyang’s findings^18^, a Pr value for Alg-Gel bioink in the range of 0.9–1.1 indicates that the 3D-printed hydrogel construct l exhibits excellent filament morphology and mechanical stability. According to (Fig. 7a), the Pr value of Alg-Gel-GO_1_ was 0.85 ± 0.06, which is the highest viscosity. Our findings indicate that adding GO to the bioinks significantly enhanced the shape fidelity and resolution of the 3D-printed scaffolds, primarily because of the quicker viscosity recovery after the bioink was extruded^10^. Alg-Gel-PRP_0_ and Alg-Gel-PRP_1_ were printable, with Pr values of 0.83 ± 0.01 and 0.8 ± 0.04, respectively, and produced smooth, cohesive grid structures. In contrast, the grids of Alg-Gel-PRP_2_ exhibited a crooked and uneven shape. Although Alg-Gel-PRP_2_ with a Pr value of 0.76 ± 0.04 was printable at 25 °C, it lacked sufficient viscosity to preserve the grid structure. In this regard, the concentration of PRP (0%, 1%, and 2% w/v) and its proportion were selected based on the printability of the inks in our experiment (Fig. 7b).

The degradation of the scaffold caused greater cell exchange among the in-situ autologous tissue and the scaffold, allowing more accurate delivery of the seeded cells or bioactive molecules to the damaged site. Thus, the degradation behavior of biomaterials is an essential factor for TE applications^43,44^. The results showed that all scaffolds were biodegradable and that the weight loss of the groups increased over time and decreased with the incorporation of PRP in Alg-Gel scaffolds. The formation of a fibrin network within the constructs, driven by the crosslinking of fibrinogen in PRP, could explain this phenomenon^45^. In the biodegradation analysis, the degradation rate of free-PRP scaffolds over 28 days was relatively higher than that of PRP scaffolds, and the mass loss percentage was 78%. With the addition of PRP, the mass loss percentages decreased to 60% and 64% for groups of Alg-Gel-GO-PRP_1_ and Alg-Gel-GO-PRP_2_, respectively. In an investigation by Zhao et al.^43^, an increase in the PRP concentration increased the remaining mass percentage of Alg-Gel composite hydrogel bioinks. They also exhibited a slower degradation rate in groups with higher PRP concentrations than in those with lower PRP concentrations. Figure 7c illustrates that the Alg-Gel-GO-PRP_2_ scaffold degrades slightly more quickly than Alg-Gel-GO-PRP_1_, largely due to lower crosslinking and the formation of a structure that allows for greater water uptake, resulting in increased degradability. However, Alg-Gel-GO-PRP_1_ and Alg-Gel-GO-PRP_2_ demonstrated relatively similar degradation profiles, and no noticeable difference in weight loss was perceived within soaking days. The in vitro degradation rate of all scaffolds was > 4 weeks, supporting the regeneration of cartilage^45^.

Mechanical tests revealed that the compressive strength of the triphasic scaffolds increased with the incorporation of PRP (Fig. 7d). The compressive strength of the 3D-printed scaffolds with PRP (1.15 MPa for Alg-Gel-GO-PRP_1_ and 1.01 MPa for Alg-Gel-GO-PRP_2_) was higher than that of the scaffolds without PRP (0.88 MPa), which was in line with the results of the degradation study^45^. The mechanical strength of Alg-Gel-GO-PRP_2_ is slightly lower than that of Alg-Gel-GO-PRP_1_, but the difference is insignificant. The ultimate compressive strength of cancellous bone ranges from 0.7 to 30 MPa, with cellular sawbones exhibiting values between 1.4 and 5.4 MPa and trabecular bone structures ranging from 0.7 to 30 MPa^46^. The compressive strength of the triphasic scaffold fulfilled the requirements of a porous scaffold for OC regeneration.

The frequency sweep test showed that the storage modulus (G’) value was higher than the loss modulus (G″) value in the whole angular frequency range in both hydrogels (Fig. 7e), demonstrating that the elastic property dominated the viscous property while applying a load. This behavior indicates typical gel structure formation, characterized by a solid-like behavior in hydrogels^45,47^. The storage modulus was around 630 Pa in the Alg-Gel-PRP_1_ hydrogel, which was relatively higher than that of the PRP_2_ hydrogel (570 Pa) at the frequency of 10 s^− 1^, but a significant difference was not observed between Alg-Gel-PRP_1_ and Alg-Gel-PRP_2_. This may be due to an ionic imbalance inside the hydrogel structure followed by the incorporation of PRP; therefore, a decrease in stability was observed in the hydrogel. Furthermore, the Gʹ amount is associated with the crosslink density: the higher the Gʹ of the hydrogel, the greater the crosslink density^40,48^. Accordingly, the higher Gʹ of Alg-Gel-PRP_1_ compared to Alg-Gel-PRP_2_ may be due to the higher cross-linking density of Alg-Gel-PRP_1_ compared toAlg-Gel-PRP_2_. The thixotropic or shear-thinning nature of the hydrogels is shown in Fig. 7f, as the viscosity decreased with time under a constant shear rate of 100 s^− 1^. This property is especially beneficial for facilitating the extrusion of considerably viscous hydrogels through the printing nozzle.

#### In vitro biocompatibility analysis of 3D-printed triphasic scaffolds

**Fig. 8:**
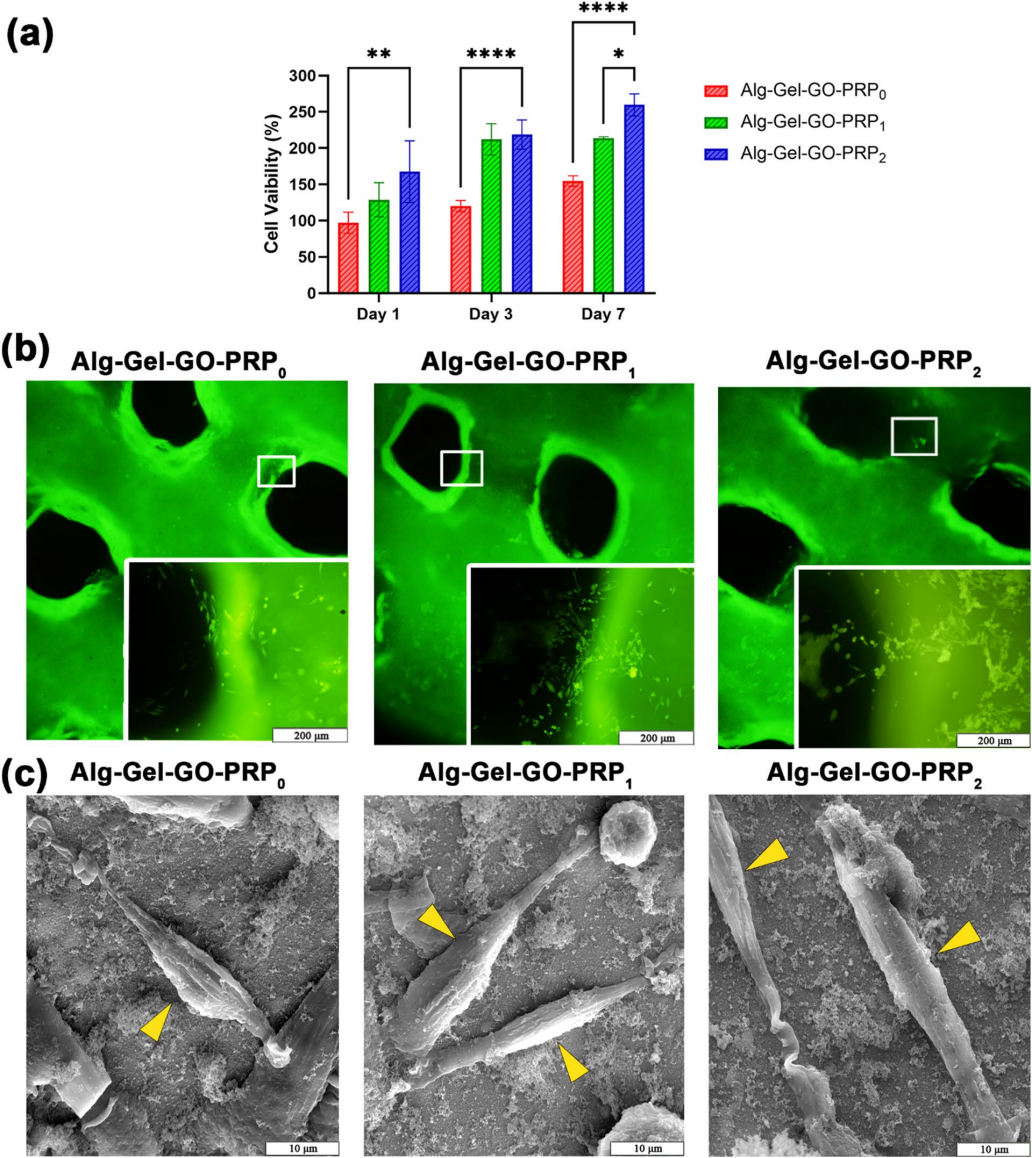
Evaluation of stem cell viability. (**a**) MTT assay for determining cell viability in 3D-printed triphasic scaffolds; (ns, no significant difference; *n* = 3, ^*^p ≤ 0.05, ^**^p ≤ 0.01, *** *p* ≤ 0.001, **** *p* ≤ 0.0001, means ± SD (**b**) Live-dead assay of BM-MSCs in different 3D-printed triphasic scaffold groups (scale bar = 200 μm). (**c**) FE-SEM images showing the adhesion of stem cells on the surface of different 3D-printed triphasic scaffold groups. The cells were well-attached to the different 3D-printed triphasic scaffold groups (scale bar = 10 μm).

To evaluate the viability of BM-MSCs seeded on 3D-printed triphasic scaffolds with various PRP concentrations, MTT assays were performed. The results showed that on days 3 and 7, Alg-Gel-GO-PRP scaffolds with 1% PRP and 2% PRP had significantly higher cell proliferation compared to the 0% PRP (Fig. 8a). PRP offers a combination of natural growth factors and a hydrated extracellular matrix-like microenvironment that enhances cellular viability and proliferation. PRP promotes cellular proliferation and decreases cell death through inhibiting Bcl-2 expression and apoptosis^49,50^. These findings are consistent with those of previous studies showing enhanced cellular metabolic activity and proliferation with the addition of PRP^51^. For example, Singh et al. demonstrated that cells encapsulated within sodium alginate (SA), chitosan/chondroitin sulfate (PEC), silk fibroin (SF), and PRP scaffolds for cartilage tissue regeneration exhibited noticeably higher metabolic activity and proliferation rates compared to PEC/SF and PEC/SF/SA scaffolds^49^. However, Choi et al.^52^ reported decreased cell viability and proliferation at higher PRP concentrations. Similarly, Tavassoli-Hojjati et al.^53^ found that 5% PRP had the most significant effect on fibroblast proliferation after 3 days, and increasing the PRP concentration by up to 50% reduced cell viability and proliferation. Therefore, Alg-Gel-GO-PRP_2_ bioink was found to be an optimal biomaterial to regenerate OC tissue and showed encouraging in vitro results toward that goal. Figure 8b shows live/dead staining performed 72 h after stem cell seeding on the scaffolds, highlighting numerous viable cells (green) and minimal presence of dead cells (red) across all groups. The different concentrations of PRP (0%, 1%, and 2% w/v) showed no obvious difference in cell viability, indicating great biocompatibility for all 3D-printed triphasic scaffolds and no obvious cell mortality. In addition, the proliferation rate of viable cells at 2% PRP was higher than that in the other groups (Fig. 8b). Dead cells did not show an increase in the cell viability images at 72 h and were likely degraded. All groups supported cell attachment and proper morphology. However, the triphasic scaffolds with 2% PRP exhibited superior cell spreading and cell-cell interactions after 72 h of culture. An increase in the concentration of PRP enhanced cell-spreading capabilities, resulting in the homogeneous dispersion of stem cells with spindle-shaped morphologies. Stem cell attachment was assessed using FE-SEM after 48 h (Fig. 8c). Findings showed that the triphasic scaffold with 2% PRP promoted favorable conditions for cell spreading and elongation, as PRP GFs like PDGF and TGF-β enhance cytoskeletal organization, improving cell-matrix interactions and overall morphology^54^.

#### In vitro chondrogenic differentiation of BM-MSCs

**Fig. 9:**
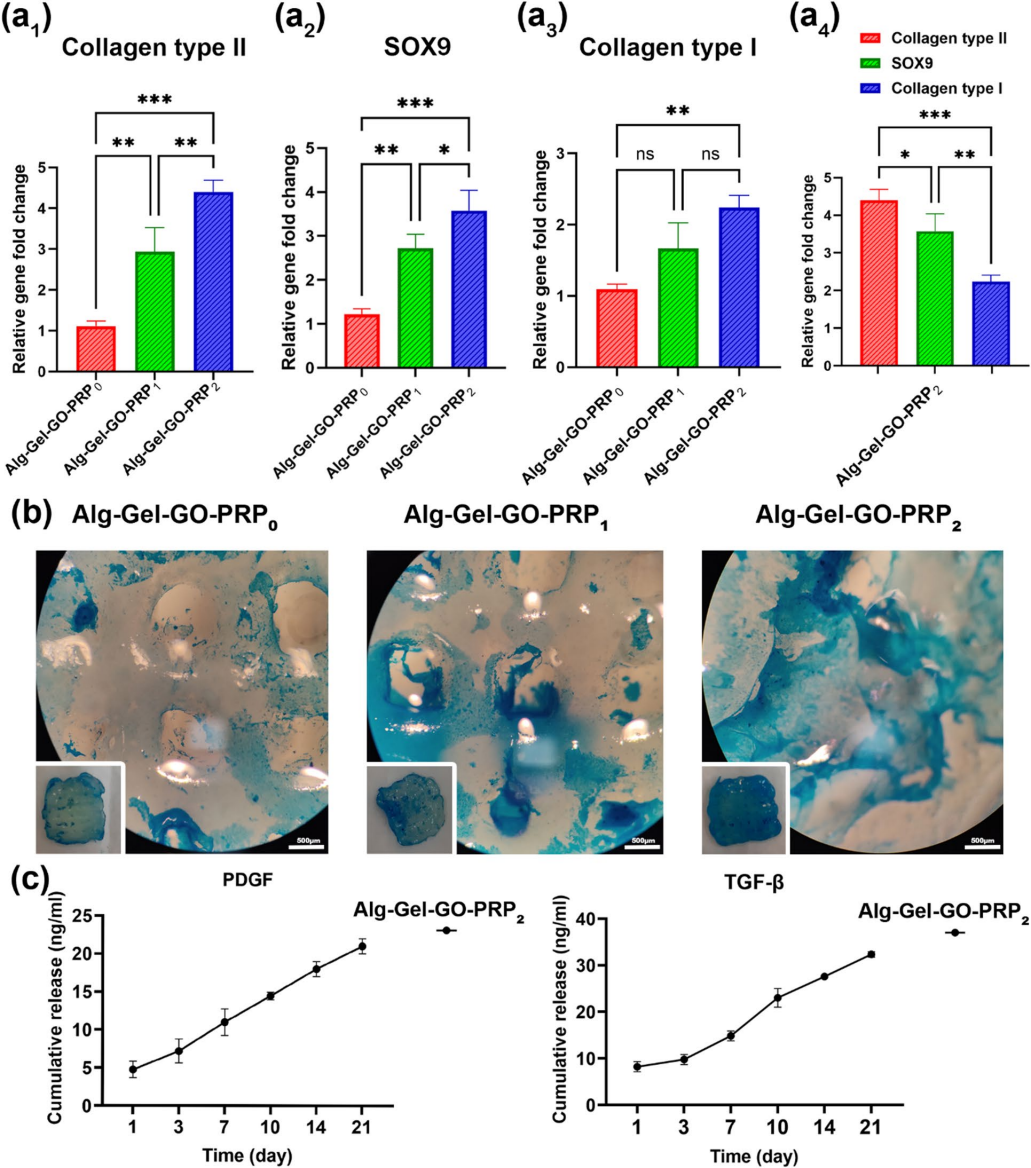
(**a**_**1–4**_) Gene expression levels of chondrogenic markers (collagen type II, SOX9) and fibrocartilage marker (collagen type I) in different triphasic scaffold groups by BM-MSCs. The values are presented as fold changes and normalized against the GAPDH reference value (*n* = 3); ns, no significant difference; ^*^p ≤ 0.05, *** *p* ≤ 0.001, **** *p* ≤ 0.0001. (**b**) Alcian Blue staining of BM-MSCs following 3 weeks of chondrogenic differentiation on the surface of the triphasic scaffolds. (**c**) In vitro cumulative release of PDGF and TGF-β1 from PRP-containing scaffold  (scale bar = 500 μm).

The effect of PRP concentration on the chondrogenic differentiation of BM-MSCs on 3D-printed triphasic scaffolds was estimated by measuring the expression of collagen type II and SOX9, the cartilage-specific genes, along with bone-specific genes like collagen type I (Fig. 9a_1−3_). The RT-PCR analysis revealed that the 3D-printed triphasic scaffold groups containing 2% PRP strongly upregulated the chondrogenic markers SOX9 and collagen type II compared with the other groups (**P ≤ 0.01 with 1% PRP, ***P ≤ 0.001 without PRP). Previous research indicated that a high concentration of 10% PRP in the differentiation medium could induce adipose-derived MSCs to differentiate into chondrocytes^55^. In addition, lyophilized PRP powder plays remarkable roles as a storage vehicle for GFs, including TGF-β and PDGF, as demonstrated by ELISA tests. Our research demonstrated that the incorporation of PRP into a scaffold facilitates the sustained release of growth factors for a duration of up to 21 days. TGF-β and PDGF are well known to induce MSC chondroblast differentiation and the accumulation of cartilage ECM^56^. Conversely, the mRNA level of collagen type I (a positive fibrocartilage marker) was downregulated in the triphasic scaffold group with 2% PRP compared with other chondrogenic markers like collagen type II and Sox9. A previous study demonstrated that adding 5% PRP to the culture medium of adipose-derived MSCs enhances osteogenic marker expression^57^. However, our research indicates that incorporating 2% PRP in a 3D-printed triphasic scaffold group increases chondrogenic marker expression more than osteogenic marker expression (***P ≤ 0.001) (Fig. 9a_4_). Moreover, the in vitro chondrogenesis of BM-MSCs in the 3D-printed triphasic scaffold was also figured out by histological evaluation of ECM deposition with Alcian Blue staining (Fig. 9b). With increasing GF concentration, the PRP-loaded scaffold groups exhibited greater positive staining for Alcian Blue in the matrix surrounding the stem cells. Alcian Blue staining revealed that 2% PRP in the 3D-printed scaffolds increased staining intensity, indicating higher GAG content in the ECM around the stem cells compared with the other groups^49^(Fig. 9b). Notably, BM-MSCs on scaffolds with 2% PRP exhibited noticeably enhanced chondrogenic gene expression and cartilage-like ECM. In-vitro release tests of TGF-β1 and PDGF growth factors from a PRP-containing scaffold were conducted over 21 days, with the release profiles illustrated in Fig. 9c. A burst release occurred within the initial 24 h. The release of growth factors persisted in a regulated manner for 21 days thereafter.

Our findings showed that 2% PRP improved cell responsiveness and effectively promoted chondrogenic differentiation, along with cartilage ECM regeneration of BM-MSCs in a biomimetic environment.

### Study limitation

Cell source: The study used rat MSCs instead of human cells, which may limit differentiation potential. However, rat MSCs are easily accessible, raise fewer ethical concerns, and exhibit similar cell behavior. Future research will incorporate human-derived cells to improve clinical applicability.

Duration of assays: Only short-term in vitro assays were performed. Longer culture periods will be necessary to fully evaluate scaffold performance and matrix maturation.

In vivo validation: The current study did not include in vivo experiments. Animal models will be essential in future investigations to confirm biocompatibility, integration, and functional outcomes.

PRP variability: Variability in platelet-rich plasma donor composition was not assessed; however, consistent with previous studies, we pooled approximately 10 samples from randomized cases. Standardization and characterization of PRP preparations are crucial to minimize experimental variability and enhance reproducibility.

By explicitly acknowledging these limitations, we aim to provide a balanced perspective on the scope of our findings while outlining clear directions for future research.

## Conclusion

This study utilized extrusion-based multi-nozzle 3D printing to create tri-layered scaffolds that mimicked OC tissue. First, we added GO gradients to the Alg-Gel bioink as a subchondral bone layer and investigated its chemical composition, swelling, rheological and mechanical properties, and biocompatibility. The optimal concentration for printing the subchondral bone layer construct and preserving its density in the other triphasic scaffold layer was 1% GO. Then, Alg-Gel-GO_1_ bioink was used to print triphasic scaffolds with varying PRP gradients. The degradation, rheological, mechanical, and printability properties of the bioinks were further investigated, and scaffold biocompatibility was tested in vitro using BM-MSCs. We found that all bioinks increased BM-MSCs’ chondrogenic development after 3 weeks in chondrogenic media. The bioink composed of Alg-Gel-GO-PRP_2_ was the best for chondrogenic gene expression and GAG deposition. According to our experiment, the 3D-printed Alg-Gel-GO-PRP_2_ is a good option for OCTE due to its ECM-mimicking and biocompatibility. Our findings show that 3D-printed OC scaffolds with triphasic structures promote chondrogenesis and have considerable potential for OC regeneration.

## Data Availability

The datasets generated during and/or analysed during the current study are available from the corresponding author on reasonable request.
